# Effect of Indolic-Amide Melatonin on Blood Cell Population: A Biophysical Gaussian Statistical Analysis

**DOI:** 10.3390/molecules23061378

**Published:** 2018-06-07

**Authors:** Roberto Zivieri, Fabio Borziani, Angela Strazzanti, Angela Fragomeni, Nicola Pacini

**Affiliations:** 1 Department of Mathematical and Computer Sciences, Physical Sciences and Earth Sciences, University of Messina, 98166 Messina, Italy; 2 Laboratory of Biochemistry F. Pacini, 89100 Reggio Calabria, Italy; info@fabioborziani.it (F.B.); pacini.biochemistry@gmail.com (N.P.); 3 Department of General Surgery and Senology, University Hospital Company, 95124 Catania, Italy; angelastrazzanti@yahoo.it; 4 Laboratory of Analysis Chemical Clinical and Section of Hematology and Coagulation, Provincial Health Company, 89100 Reggio Calabria, Italy; afrag@hotmail.it

**Keywords:** indolic compounds, melatonin, placebo, leukocytes circadian variations, leukocytes, hematology, differential diagnosis, statistical analysis, Gaussian distribution

## Abstract

The problem of the correlation of indolic molecules with special regard to melatonin and immune processes has been widely investigated. However, there are only few studies focusing on circadian variation of peripheral blood leukocytes. The purpose of this study is thus to understand the influence of MLT on leukocyte populations and its correlation with leukocyte distribution. This is accomplished by administrating placebo and melatonin to different groups of individuals and by performing a biophysical Gaussian analysis on the number of leukocytes by means of a comparison of their p.m. vs. a.m. variations under the effect of placebo and of melatonin and via a comparison in the morning between leukocytes population of untreated group and MLT group. It is shown that: (a) melatonin has the effect of narrowing the normal distribution concentrating most of the individuals towards the mean value of the observed variation of leukocytes population and (b) the individuals who have not received either placebo or supplement have a leukocyte population that follows a normal distribution. These results confirm the crucial role played by melatonin, as the most representative of indolic amide in biological systems, in the circadian peripheral variations of leukocyte numbers because counts of white blood cells are essential in medical urgency and differential diagnosis situations. Hence, further studies are suggested to account for these physiological variations and for the evaluation of the full involvement of the action of MLT on leukocytes distribution.

## 1. Introduction

It is well-known that many biological phenomena occurring in living systems are subject to periodical variations in amplitude and frequency [1]. As a result, there is the existence of an internal time regarded as a characteristic time. There have been extensive investigations on both the physiological and the molecular aspects of this fact. In this respect, examples are reproductive cycles with seasonal and monthly rhythms in women, circadian variations of blood pressure and of temperature, hormonal peak of cortisol and so on. In addition, it has been observed a circadian variation in many biochemical pathways such as, e.g., the glycolysis pathway and the synthesis of mRNA [2,3,4,5]. 

To fully understand this phenomenon it should be taken into account that most metabolic reactions exhibit autocatalytic patterns regulated by feedback mechanisms able to generate a given internal frequency [6,7].

However, only a few studies have been devoted to the investigation of circadian variations of the emo-cytometric composition of peripheral venous blood. Moreover, in those works, only a small number of subjects were employed and this limitation has led to partially contradictory results. All this occurred even though the study of the corpuscular component of the venous peripheral blood and of its physical-chemical features represents an important instrument in clinical biochemistry, especially in medical urgency and differential diagnosis situations. 

In 1956 Brown and Dougherty [8] studied the circadian variations of the corpuscular fraction of venous peripheral blood showing inconclusive data. Subsequently, Richens et al. [9] investigated the circadian variations of the rates of leukocytes, cortisol and in vitro the migratory capacity concluding that there was an acrophase for the number of leukocytes between 8 p.m. and midnight followed by a nadir between 8 a.m. and noon where the migratory capacity was maximum corresponding to three hours after the cortisol peak. On the other hand, Bertouch et al. [10] have shown that the rate of circulating leukocytes has an acrophase between 6 p.m. and 10 p.m. exhibiting a maximum value at 9 p.m. and a nadir at 9 a.m. Similar results have been reported by Ritchie et al. [11]. More recently, Sennels et al. [12] have found an acrophase for leukocytes between 9 p.m. and midnight (with a mean value at about 9 p.m.) followed by a progressive decrease at about 3 a.m. and a nadir at 9.a.m. In this work, melatonin (MLT) levels have also been determined, indicating an acrophase at about 3 a.m. and a nadir at about 9 a.m. marking a role in the regulation mechanism of the rate of circulating leukocytes.

As it is well-known MLT secreted from the pineal gland is strongly connected with the regulation of circadian rhythms, through a complex nervous circuit that starts from retinal photoreceptors with a serotoninergic modulation mediated in the suprachiasmatic nucleus and fibers afferent to the superior cervical ganglion. From this ganglion originates the innervation to the pineal gland giving rise to a connection among the retinoic photoreceptors, suprachiasmatic nucleus and pineal gland itself. Indolamide MLT has been linked to many biological effects such as regulation of apoptosis, regulation of oxidative phosphorylation, Reactive Oxygen Species homeostasis, cytoskeletal function and antiproliferative effects [13]. 

Likewise, despite the fact that in several studies a correlation of the MLT level with immune processes and the immune-hematopoietic system has been shown, there are only a few studies on chemotaxis and leukocyte diapedesis. Moreover, even though standard conditions offer a valid basis of reproducible samples employed in these studies, they could be far from the real conditions in healthy subjects under the influence of working activities [14,15,16,17,18,19,20]. Note that also some recent studies on white blood cells (WBCs) and their efficient adhesion to the vascular endothelium and on red blood cells (RBCs) aggregates in the form of rouleaux, due to the presence of plasma proteins, and on RBCs shapes have been carried out to understand the role of these blood populations [21,22,23].

On this basis, the aim of this work was to provide an estimate of the nadir and of the acrophase of leukocyte populations and to establish the existence of a possible involvement of MLT in the regulation of the levels of circulating leukocytes. We have analyzed the experimental results via a simple statistical analysis confirming that the WBCs population under the effect of MLT follows a Gaussian distribution. Note that in the literature there are powerful statistical methods like, e.g., ANOVA and cosinor applied to time-periodic patterns by means of which it is possible to determine the statistical parameters of a population [24]. However, for our purposes, we have preferred to perform an original approach based on the classical normal distribution able to give a direct response about the effect of MLT on leukocytes population during the 12 h. If we wanted to perform a quantitative evaluation of a total circadian variation, we would have used the common methods of the literature. In other words, in this context, our aim was the determination of the mean amplitude of leukocytes population during an interval of 12 h that could exhibit an acrophase at the beginning and a nadir at the end. However, we would like to remind that, for our aims, it was not important that the maximum and the minimum of leukocytes are in this temporal window because we wanted to test the possible effects of MLT on leukocytes distribution.

## 2. Results

In this section, we present the main results of the statistical Gaussian analysis carried out on the individuals chosen in the experiment performed to evaluate the effect of MLT on the number of leukocytes. The mathematical details of the statistical analysis are described in Section 4 (Materials and Methods). 

We have randomly selected a group of 103 individual (49 males and 54 females) with ages ranging between 21 and 65 years old with an average age of 44 years old (see Table 1). Each volunteer gave consent. Each individual was healthy, did not take drugs and alcohol and followed a Mediterranean diet. Night rest time was not inferior to 7 h and not superior to 8 h from 23 p.m. to 8 a.m. Moreover, since physical exercise could influence chemiotaxis and diapedesis, individuals performing arduous work or practicing competitive sports were excluded. In Table 1 we summarize the demographic data for all participants with Body Mass Index (BMI). The MLT was prepared according to the specifications of the European Pharmacopoeia PhEu.

Table 2 summarizes variations of hemocytometric parameters in placebo group of 24 individuals and includes also t-Student statistics where *p* stands for probability. Here, PLTs stands for platelets, HG for hemoglobin, HCT for hematocrit, MCV for corpuscular volume. Interestingly, there is a decrease of WBCs population passing from their PM values to their AM values involving all families of leukocytes. Specifically, as should be expected, the most relevant variations that are statistically significant regard the populations of lymphocytes and neutrophils. This behaviour is not surprising because these two families have a relevant turnover that involves also chemiotaxis, diapedesis and leukocytes homing mechanisms. 

**Figure 1:**
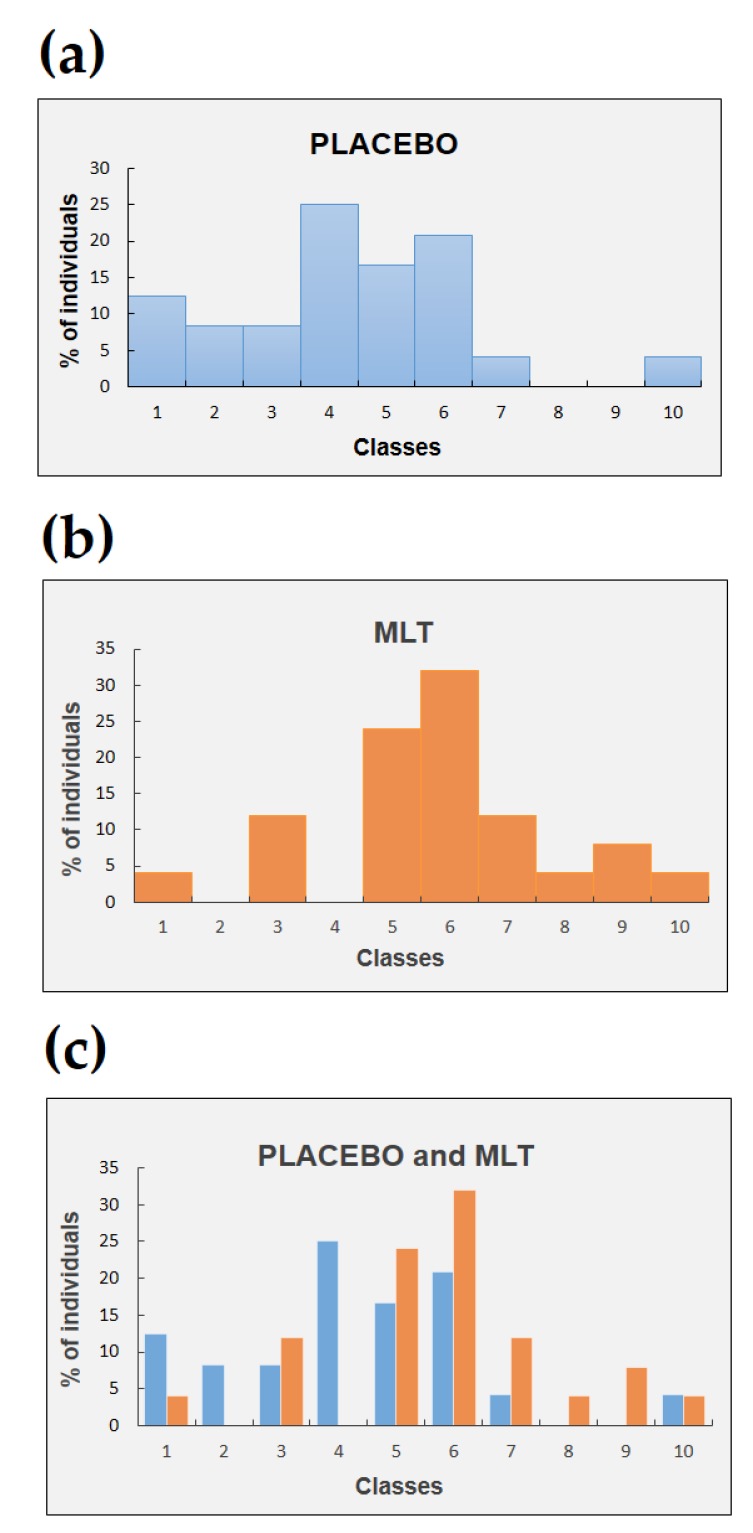
Percentage of individuals schematized by hystograms vs. classes. (**a**) Individuals (blue hystograms) who have received placebo. (**b**) As in (**a**) but for individuals (orange hystograms) who have received MLT. (**c**) Comparison between the number of individuals belonging to a given class who have received either placebo (blue) or MLT (orange). In all panels the meaning of classes in terms of leukocytes variation (in%) from the acrophase to the nadir is: 1 ⇔ positive variation, 2 ⇔ 0–5% negative variation, 3 ⇔ 5–10% negative variation, 4 ⇔ 10–15% negative variation, 5 ⇔ 15–20% negative variation, 6 ⇔ 20–25% negative variation, 7 ⇔ 25–30% negative variation, 8 ⇔ 30–35% negative variation, 9 ⇔ 35–40% negative variation, 10 ⇔ 40–45% negative variation.

**Figure 2:**
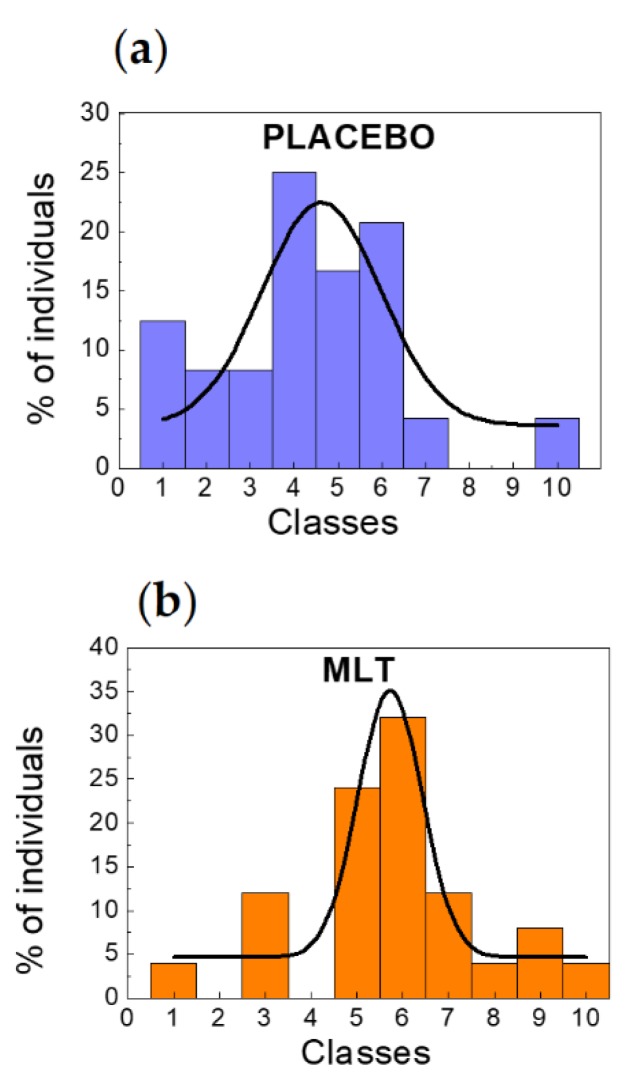
Individuals (%) vs. classes compared to a Gaussian fit distribution. (**a**) Percentage of individuals who have received placebo (blue histograms) and Gaussian distribution (black continuous curve). (**b**) As in (**a**) but for individuals who have received MLT (orange histograms). In both panels the meaning of classes in terms of leukocytes variation (in %) from the acrophase to the nadir is: 1 ⇔ positive variation, 2 ⇔ 0–5% negative variation, 3 ⇔ 5–10% negative variation, 4 ⇔ 10–15% negative variation, 5 ⇔ 15–20% negative variation, 6 ⇔ 20–25% negative variation, 7 ⇔ 25–30% negative variation, 8 ⇔ 30–35% negative variation, 9 ⇔ 35–40% negative variation, 10 ⇔ 40–45% negative variation.

In Table 3, variations of hemocytometric parameters in MLT group of 25 individuals are shown together with a t-Student statistics. Note that the variations of WBCs population are bigger with respect to those of the placebo group for all families with the exception of eosinophils. The bigger average variation of leukocytes for MLT group reflects the significant variations pushed towards the higher classes (see Figure 1 and Figure 2). Finally, although variation of eosinophils is not statistically significant for both groups, it is interesting to note that the minor variation regards the MLT group. This minor variation could be attributed to the action of MLT on ACTH and cortisol signaling. 

First, we describe the measurement of half-circadian variation of leukocytes population and then the non-circadian one. Concerning the evaluation of nadir vs. acrophase of placebo vs. MLT, although there are only a few studies on the topic, they all agree on indicating an average leukocyte acrophase at about 9–10 p.m. and a nadir between 8.30 a.m. and 9 a.m. A first group of 49 individuals was subdivided into two subgroups. Both the group of 24 individuals who have received placebo and the group of 25 individuals who have received a MLT supplement before venous sampling were subjected to a venous sampling from the cubital vein of the arm between 9 p.m. and 9.30 p.m. followed by a venous sampling by means of a vacutainer system in the same temporal order between 9 a.m. and 9.30 a.m. of the following day. We have administered placebo to the first subgroup and 1 mg/kg of bw, of MLT in gastro-resistant formulation to the second subgroup and the individuals ignored whether they assumed either one or the other substance.

Concerning the 72 h non-circadian evaluation, a further 54 untreated individuals (28 males and 26 females) were subject to a blood extraction from the cubital vein of the arm between 9 a.m. and 9.30 a.m. and the measured leukocyte values were taken as reference mean values in the morning. Table 4 shows the hemocytometric parameter comparison of 54 individuals of the untreated group and the corresponding ones of the 25 MLT group individuals subjected to a venipuncture from the cubital vein of the arm between 9 a.m. and 9.30 a.m. We also show variations between the parameters in the two groups. Remarkably, the group treated with MLT exhibited a significant morning WBC population reduction not only with respect to the placebo group (see Table 2 and Table 3) but also with respect to the untreated group. This trend could suggest that a supraphysiological dose of MLT (1 mg/kg of bw) strongly reduces the leukocyte population. 

Subsequently, the group of 54 individuals has then been randomly subdivided into three subgroups of 12, 20 and 22 individuals, respectively. We have given placebo to the first subgroup for two subsequent evenings, 0.1 mg/kg of bw of MLT to the second subgroup and 1 mg/kg of bw of MLT to the third subgroup. Also in this case the individuals ignored whether they assumed either one or the other substance. After 3 days and three administrations at a temporal distance of 72 h, all the individuals were subject to a venipuncture from the cubital vein of the arm between 9 a.m. and 9.30 a.m. We have then collected the blood in test tubes with EDTA and we have analyzed it using an Advia 120 hemochromocytometric appliance (company, city, state abbrev if USA, country). 

It is particularly interesting to note that, when the measurement is performed comparing the morning to morning parameters like in these subgroups, there are no significant statistical variations in the blood cell populations. This behaviour could indicate two things: (1) the observed variations regard a circadian variation and (2) the shown action of MLT could not be mediated by a direct action of hematopoiesis. 

Figure 1 shows the number of individuals (in percentage) represented in term of histograms for both subgroups (placebo and MLT) as a function of the time variations of the leukocyte population. 

The number of individuals who received a placebo were 24, while the ones who received MLT were 25. Each class corresponds to a variation of 5% of the number of leukocytes from the acrophase in the evening to the nadir in the morning. In particular, number 1 refers to the class corresponding to a positive variation, number 2 to a negative variation ranging between 0 and 5%, number 3 to a negative variation ranging between 5% and 10% and so on with the last class corresponding to a negative variation ranging between 40% and 45%. For the placebo subgroup, there is a concentration of the number of individuals in the first classes. Instead, for the MLT subgroup the tendency is towards a concentration of individuals in the last classes corresponding to the strongest variations of leukocyte number.

As depicted in Figure 2, the number in percentage (%) of individuals (red histograms) who have received placebo and MLT as a function of circadian variations of leukocytes can be represented in terms of a Gaussian distribution (black continuous curves). Interestingly, the effects of MLT are: (1) to shift the maximum amplitude and therefore the mean value towards higher values corresponding to higher classes and (2) to narrow the distribution in agreement with the central limit theorem. Note that the numbers resulting from the fit that are discussed in the following are not absolute numbers but are referred to%. A quantitative analysis gives a shift of the mean value of the Gaussian distribution from 4.62 to 5.72 (from 10–15% to 15–20% leukocytes negative variation) passing from placebo to melatonin. Moreover, we have obtained a standard deviation σ = 1.36 for individuals who have received placebo (Figure 2a) corresponding to more than 5% leukocytes negative variation to σ = 0.70 for individuals who have received MLT (Figure 2b) corresponding to less than 5% leukocytes negative variation. In addition, to confirm this trend, the full width at half-maximum (FWHM) Γ is Γ = 3.19 for individuals who have received placebo and Γ = 1.65 for individuals who have received MLT. 

To summarize, passing from placebo to MLT supplement, there is a shift of the mean value of about one class, a reduction of σ of about 50% and a FWHM halving. In addition, we have calculated, according to Equation (1), Section 4 (Materials and Methods), the area *Area*_[−σ, σ]_ subtended by the Gaussian curve in the interval [−σ, σ] getting: *Area*_[−σ, σ]_ = 43.81 with a maximum amplitude corresponding to the mean value *C* = 64.17 for individuals having received placebo and *Area*_[−σ, σ]_ = 36.63 with a maximum amplitude *C* = 53.65 for individuals having received MLT. Note that the total amplitude in Figure 2a,b includes also a *y* offset not included in the maximum amplitude *C*. Correspondingly, the complementary areas subtended by the distributions outside the first standard deviation are *Area*_[−∞, −σ]_ + *Area*_[σ, +∞]_ = 20.36 for placebo case and *Area*_[−∞, −σ]_ + *Area*_[σ, +∞]_ = 17.02 for MLT case. 

This statistical analysis shows the narrowing effect of MLT highlighted by the reduction of the standard deviation and of both the area subtended by the curve within the first standard deviation and the complementary area together with the tendency towards higher leukocyte variations in the presence of MLT. This is one of the key results of this study, confirming the important role played by MLT in the regulation of the number of blood population with special regard to leukocytes population. From this analysis, it turns out that MLT causes an increase of the variation and tends to concentrate these variations towards the class having a higher number of individuals represented in this description by the 6th class corresponding to a 20–25% negative variation of leukocytes population from the acrophase to the nadir.

**Figure 3:**
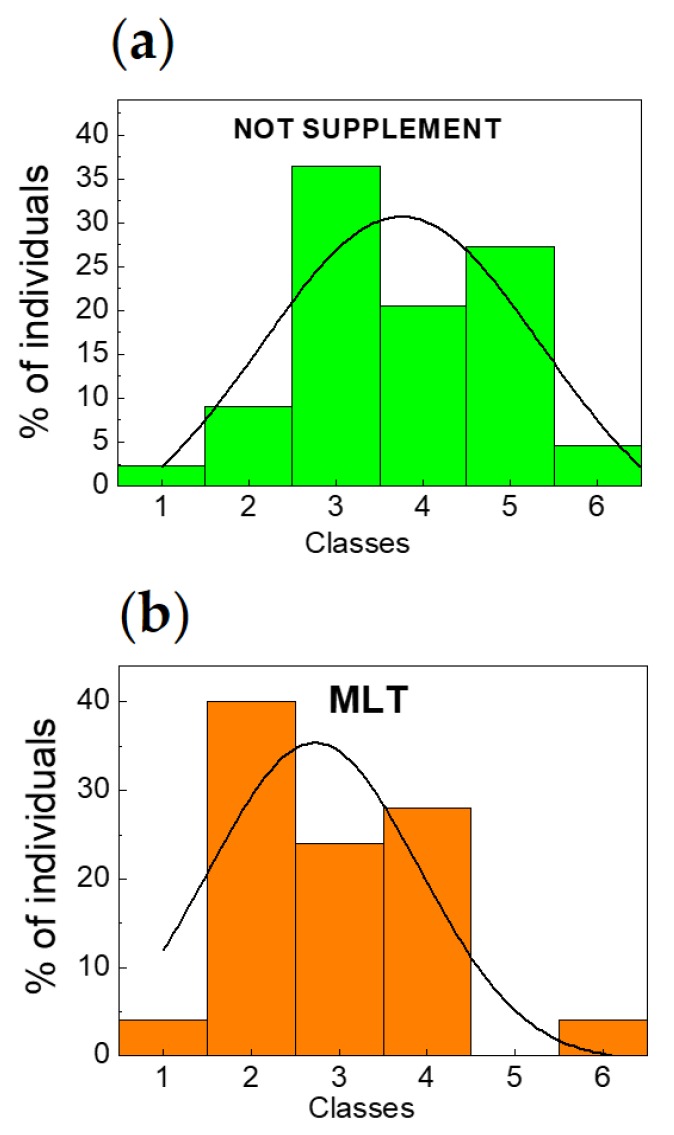
Individuals (**%**) vs. classes compared to a Gaussian fit distribution. (**a**) Percentage of individuals who have not received supplement (green histograms) vs. classes compared to a fitting Gaussian distribution (black continuous curve). (**b**) As in (**a**) but for individuals who have received MLT (orange histograms). In both panels the meaning of classes in terms of number of leukocytes is: 1 ⇔ 4000–5000/µL, 2 ⇔ 5000–6000/µL, 3 ⇔ 6000–7000/µL, 4 ⇔ 7000–8000/µL, 5 ⇔ 8000–9000/µL, 6 ⇔ 9000–10,000/µL.

In Figure 3a, we display the number of individuals for the group of 54 subjects who have not received supplement (in other words, not treated) as a function of leukocytes population measured in the morning. This statistical analysis is compared to the one performed on the 25 subjects who have received MLT (Figure 3b). Note that the 25 subjects who have received MLT are the same individuals whose statistics is shown in Figure 2b but in this case only one hemocytometric measure in the morning has been performed. Among the 54 subjects, we have excluded 10 subjects having pathological values of leukocytes (>10,000/µL). Hence, the statistical analysis of this group has been carried out on 44 subjects. The important result is that the leukocytes distribution of the 25 subjects treated with MLT is statistically different with respect to the one of the 24 placebo subjects p.m. a.m. and the 54 subjects who have not received a supplement. We would like to remind readers that, for the 54 subjects, the 72 h test did not give any significant statistical variation. This suggests that the MLT effect could not be mediated by a direct action of hematopoiesis at the level of bone marrow. 

Both the subjects who have not received supplement (Figure 3a) and the ones who have received MLT (Figure 3b) were divided into classes each one of 1000/µL leukocytes concentration starting from 4000/µL to 10,000/µL. It is evident, from the statistical analysis (black lines) that also for this group there is a narrowing effect due to the MLT. However, unlike for the previous case, MLT shifts the maximum amplitude and the mean value of the Gaussian distribution from the 4th (7000–8000/µL) to the 3rd class (6000–7000/µL), namely towards a lower class corresponding to a lower number of leukocytes. In particular, for the individuals who have not received supplement, we have found, by means of the statistical analysis, the following fitting parameters: σ = 1.61 corresponding to a variation of the number of leukocytes between 1000/µL and 2000/µL, Γ = 3.79 and *Area*_[−σ, σ]_ = 102.03 with a maximum amplitude *C* = 149.45 in correspondence of the mean value. On the other hand, for individuals who have received MLT, we have found σ = 1.19, Γ = 2.80 and *Area*_[−σ, σ]_ = 73.15 with a maximum amplitude *C* = 107.15 in correspondence of the mean value. The complementary areas are *Area*_[−∞, −σ]_ + *Area*_[σ, +∞]_ = 47.42 for placebo case and *Area*_[−∞, −σ]_ + *Area*_[σ, +∞]_ = 34.00 for MLT case. Also in this case the subtended areas for the case of MLT are smaller with respect to the ones of the case of subjects who have not received supplement. For the mathematical and statistical tools used for this analysis, see Section 4 (Materials and Methods).

## 3. Discussion

Recently, it has been shown in healthy subjects that sleep deprivation was related to an increase of the number of circulating leukocytes and to several metabolic variations [25]. More recently, Ackermann et al. and Boudjeltia et al. [26,27] have confirmed that sleep deprivation has a strong influence on the number of leukocytes with special regard to granulocytes eliminating patterns of circadian modifications in the differential count. Note that there is a strong link between sleep deprivation and destruction of the circadian rhythm of MLT secretion with decreased MLT plasma levels.

Our results show a broad variation in various cellular elements of peripheral blood with special regard to the total number of leukocytes and of all their fractions like monocytes, lymphocytes and granulocytes. In particular, there is a strong decrease in the morning of most of the populations with the exception of eosinophil granulocytes exhibiting increasing values. In accordance with previous studies on sleep deprivation, in which there is the absence or a strong reduction of MLT secretion, our analysis, with results opposite to those of sleep deprivation, shows that MLT strongly strengthen this effect indicating an active role in circadian regulation of corpuscular composition of peripheral venous blood. Besides a circadian variation of the number of circulating leukocytes, in the literature there have been studies reporting a circadian variation of the number of bone marrow blasts having an opposite trend, namely with an acrophase for the number of myeloid precursors at about 9 a.m. and a nadir in the evening with a prevalence of a S phase during the night [28]. 

Since we have not obtained important variations in the 72 h test in subjects with MLT and placebo, it is reasonable to attribute the relevant effects due to MLT in 12 h PM vs. AM tests to its action on the circadian distribution of leukocytes, namely on the mechanisms of chemiotaxis, diapedesis and homing and not to a direct action on the red bone marrow hematopoietic system. 

In this respect, the roles of integrin-β2 (or CD18) and of integrin α (or CD11) and of the adhesion molecule I-CAM1 (or CD54) are very important. In “in vivo” models it has been shown that MLT can reduce the expression of CD18 and CD11 reducing the adhesion and the diapedesis, while in models of allergic encephalomyelitis (EME) the most important model to reproduce multiple sclerosis, MLT has shown a down-regulation of the expression of CD54 [29,30].

However, these effects have been highlighted with strong medicine doses of MLT (5–10 mg/kg of body weight (bw)) and for prolonged periods. Paradoxically, the antagonist of the MT-1/MT-2 receptor luzindole has shown a significant reduction of the progression of EME [31]. On the other hand, recent data in models of zebrafish suggest that the MLT strongly stimulate the leukocyte infiltration via a stimulation of the TNF-α and of the IL-8 [32]. However, also in this case, previous studies have contradictory and opposite results [33,34].

This apparent discrepancy is probably related to the different dose and to the different profile of receptor expression. Indeed, leukocytes express both receptor families MT-1 and MT-2 and receptor MT-3 or quinone reductase 2. Moreover, it has been suggested the heterodimerization of receptors MT-1 and MT-2 or heterodimerization with the orphaned GPCR50 receptor leading to a very heterogeneous framework [35]. Yet, it is improbable that the described mechanisms are responsible of the observed effects because the regulation of the CD18, CD11 and CD54 gene expression requires many hours and days and in any case strong medicine doses.

Cortisol has repeated circadian peaks characterized by the alternation of nadir and acrophases starting from the first hour in the morning until 10 p.m. It is interesting to note that the increase in the plasma concentration of MLT is followed by the decrease in the plasma concentration of cortisol or, in other words, the plasma concentration of MLT is inversely proportional to that of cortisol [36]. 

MLT via the bond to the GPCR-MT-1 receptor in correspondence of adrenal cortical can reduce the effects of ACTH diminishing cortisol synthesis [37,38,39,40]. Moreover, it can modulate the gene expression of receptors [41]. Cortisol peaks are associated to the liberation of IL-8 and, in turn, IL-8 leads to an increase of the number of total circulating leukocytes [42]. A mechanism, which is realized through the reduction to the response to ACTH and, in turn, to the reduction in cortisol release could partly explain the morning decrease of circulating leukocytes [43]. Note that the considerable complexity typical of these mechanisms is due to the great heterogeneity of receptors to MLT and to the differential expression of the various subtypes of these receptors in different tissues [35,36,37,38,39,40,41,42,43,44,45,46]. 

Finally, it is interesting to note that two subjects in the placebo group presented a pronounced leukocytosis in the evening and normal values in the following morning. After having performed inflammation tests, we have found that these two subjects were perfectly healthy and the same subjects did not show disease symptoms in the following days. Moreover, we underline that many biological processes are time-dependent exhibiting a time oscillatory behaviour [4,5,6,7,47,48,49]. Our results suggest the existence of a broad circadian variation of WBCs count induced by MLT and the crucial role played by MLT in the regulation of circadian variation of WBCs cells albeit within the limits of our analysis. We wish that our work could stimulate further studies to fully understand the effect of MLT on blood cells distribution. 

## 4. Materials and Methods

In this section, we present the statistical analysis performed on the data obtained through the experiment described in Section 2. First, we recall the mathematical apparatus at the basis of the analysis. 

A Gaussian or normal distribution takes the form $f\left(x,x_{M},\sigma \right)=A\;e^{-\frac{{\left(x-x_{M}\right)}^{2}}{2\sigma^{2}}}$ where $A=\frac{C}{\sqrt{2\pi }\sigma }$ is the normalized amplitude and *C* is the maximum amplitude corresponding to the mean value, σ is the standard deviation and *x_M_* is the mean value of the distribution. The area subtended by the Gaussian curve within the first standard deviation in the interval [−σ, σ] that gives the probability of finding the variable inside this interval is calculated as:

(1) $$Area_{\left[-\sigma ,\sigma \right]}=\frac{C}{\sqrt{2\pi }\sigma }\int_{x_{M}-\sigma }^{x_{M}+\sigma }e^{-\;\frac{{\left(x-x_{M}\right)}^{2}}{2\sigma^{2}}}dx=C\left(\mathrm{Erf}\left[\frac{\sqrt{2}}{2}\right]\right)$$

where Erf stands for the error function with $\mathrm{Erf}\left[\sqrt{2}/2\right]=0.68$. The area is weighted by a factor depending on the Gaussian maximum amplitude *C* times 0.68.

The probability to obtain a data outside the first standard deviation interval is given by:

(2) $$\frac{C}{\sqrt{2\pi }\sigma }\left[\int_{-\infty }^{x_{M}-\sigma }e^{-\frac{{\left(x-x_{M}\right)}^{2}}{2\sigma^{2}}}dx+\int_{x_{M}+\sigma }^{+\infty }e^{-\frac{{\left(x-x_{M}\right)}^{2}}{2\sigma^{2}}}dx\right]=C\left(\mathrm{Erfc}\left[\frac{\sqrt{2}}{2}\right]\right)$$

where Erfc = 1 − Erf is the complementary error function with $\mathrm{Erfc}\left[\sqrt{2}/2\right]=0.32$. The integral corresponding to the area outside the first standard deviation is *C* times 0.32.

## 5. Conclusions

Our experimental data strengthened by the statistical analysis show that the indolamide-compound MLT is responsible for the strong variation observed in the p.m. vs. a.m. leukocyte populations. Chemiotaxis, diapedesis and leukocytes homing mechanisms offer important targets in inflammatory and auto-immune pathologies and so signaling pathways related to MLT deserve a further study to comprehend these phenomena. Although this study was mainly focused on WBC number, the obtained results also show a significant change, albeit of smaller amplitude, for PLTs and RBCs. These data confirm the oscillatory and periodic nature of living systems. Finally, due to the importance of the strong variation shown in this analysis, this aspect should be further investigated in relation to medical emergency and differential diagnosis situations. 

We also recall that MLT is rapidly absorbed orally and undergoes a high level of first pass hepatic metabolism even though in our experiment we have used a supraphysiological dose. Regarding this, we suggest that in future studies MLT levels should be measured and a dose/response curve evaluated.

To summarize, we believe that this work: (1) is important for the number of subjects involved to study the p.m. vs. a.m. leukocyte population variation; (2) is new in terms of the statistical analysis applied to the MLT effects on variation in time of blood cells in healthy subjects under physiological conditions even though MLT has been extensively studied with respect to hematopoiesis and leukocyte counts [19].

## Figures and Tables

**Table 1:** Demographic data all participants.

Age	(44.7 ± 3.6) years
Sex	54 f - 49 m
BMI	24.3 kg/m^2^

**Table 2:** Variations (p.m. vs. a.m.) of hemocytometric parameters in placebo group.

Time	9–9.30 p.m.	9–9.30 a.m.	% Variations (t-Student)
RBCs and PLTs	Average value	Average value	
RBCs (10^6^/µL)	4.796	4.883	+1.81% (*p* > 0.05)
HG (g/dL)	13.620	13.810	+1.40% (*p* > 0.05)
HCT (%)	43.320	43.620	+0.69% (*p* > 0.05)
MCV (fL)	91.620	90.140	−1.62% (*p* > 0.05)
PLT (10^3^/µL)	261.420	255.200	−2.38% (*p* > 0.05)
WBCs	Average value	Average value	
Leukocytes (10^3^/µL)	8.944	7.786	−12.95% (*p* < 0.001)
Lymphocytes	3.018	2.675	−11.37% (*p* < 0.05)
Monocytes	0.602	0.521	−13.46% (*p* > 0.05)
Neutrophils	5.056	4.341	−14.14% (*p* < 0.001)
Eosinophils	0.200	0.186	−7.00% (*p* > 0.05)
Basophils	0.068	0.063	−7.35% (*p* > 0.05)

**Table 3:** Variations (p.m. vs. a.m.) of hemocytometric parameters in MLT group.

Time	9–9.30 p.m.	9–9.30 a.m.	% Variations (t-Student)
RBCs and PLTs	Average value	Average value	
RBCs (10^6^/µL)	4.770	4.848	+1.64% (*p* > 0.05)
HG (g/dL)	14.540	14.580	+0.28% (*p* > 0.05)
HCT%	42.130	42.580	+1.07% (*p* > 0.05)
MCV (fL)	88.770	88.420	−0.39% (*p* > 0.05)
PLT (10^3^/µL)	248.120	236.440	−4.71% (*p* < 0.05)
WBCs	Average value	Average value	
Leukocytes (10^3^/µL)	8.187	6.401	−21.81% (*p* < 0.001)
Lymphocytes	3.043	2.405	−20.97% (*p* < 0.001)
Monocytes	0.438	0.345	−21.23% (*p* < 0.001)
Neutrophils	4.393	3.366	−23.38% (*p* < 0.001)
Eosinophils	0.246	0.234	−4.88% (*p* > 0.05)
Basophils	0.067	0.051	−23.88% (*p* > 0.05)

**Table 4:** Comparison of hemocytometric parameters untreated group vs. MLT group (morning data).

Group	Untreated	MLT	% Variations (t-Student)
RBCs and PLTs	Average value	Average value	
RBCs (10^6^/µL)	4.864	4.848	−0.33%
HG (g/dL)	14.598	14.580	−0.12%
HCT%	42.622	42.580	−0.10%
MCV (fL)	88.241	88.420	+0.20%
PLT (10^3^/µL)	242.963	236.440	−2.64%
WBCs	Average value	Average value	
Leukocytes (10^3^/µL)	8.149	6.401	−21.45% (*p* < 0.001)
Lymphocytes	2.662	2.405	−9.65% (*p* < 0.001)
Monocytes	0.448	0.345	−22.99% (*p* < 0.001)
Neutrophils	4.727	3.366	−28.79% (*p* < 0.001)
Eosinophils	0.258	0.234	−9.30% (*p* > 0.05)
Basophils	0.054	0.051	−5.56% (*p* > 0.05)
